# The genome size, chromosome number and the seed adaption to long-distance dispersal of *Ipomoea pes-caprae* (L.)

**DOI:** 10.3389/fpls.2023.1074935

**Published:** 2023-03-02

**Authors:** Kangzhuo Ye, Chunxing Dong, Bin Hu, Jieyu Yuan, Jin Sun, Zixian Li, Fang Deng, Beenish Fakher, Lulu Wang, Chenglang Pan, Mohammad Aslam, Yuan Qin, Yan Cheng

**Affiliations:** ^1^ State Key Laboratory of Ecological Pest Control for Fujian and Taiwan Crops, College of Plant Protection, Fujian Provincial Key Laboratory of Haixia Applied Plant Systems Biology, Center for Genomics and Biotechnology, College of Life Science, Fujian Agriculture and Forestry University, Fuzhou, China; ^2^ Pingtan Institute of Science and Technology, Fujian Agriculture and Forestry University, Fuzhou, China; ^3^ College of Agriculture, Fujian Agriculture and Forestry University, Fuzhou, China; ^4^ Fujian Key Laboratory on Conservation and Sustainable Utilization of Marine Biodiversity, Fuzhou Institute of Oceanography, Minjiang University, Fuzhou, China

**Keywords:** *Ipomoea pes-caprae*, long-distance dispersal, genome size, chromosome number, adaption

## Abstract

*Ipomoeapes-caprae* (L.) (IPC) is a common species in tropical and subtropical coastal areas and one of the world’s most widely distributed plants. It has attracted researchers for its outstanding biological, ecological and medicinal values. It has been reported that the genetic diversity of IPCs located on different continents is very low because of their frequent gene flow. During the long journey of evolution, every aspect of the plant morphologies has evolved to the best adaptivity to the environment, seeking their survival and progeny expansion. However, the fundamental genetic characteristics of IPC and how their seed adapted to the success of population expansion remain unknown. In this study, the fundamental genetic characteristics, including the genome size and the chromosome number of IPC, were investigated. The results showed that IPC’s genome size is approximately 0.98-1.08 GB, and the chromosome number is 2n=30, providing the basic information for further genome analysis. In order to decipher the long-distance dispersal secret of this species, the fruit and seed developments, seed morphology, and seed germination were extensively investigated and described. The results showed an exquisite adaptive mechanism of IPC seeds to fulfil the population expansion *via* ocean currents. The large cavity inside the seeds and the dense tomenta on the surface provide the buoyancy force for the seeds to float on the seawater. The hard seed coats significantly obstructed the water absorption, thus preventing the seed from germination during the dispersal. Meanwhile, the fully developed embryos of IPC also have physiological dormancy. The physical and physiological characteristics of IPC seeds provide insight into the mechanism of their long-distance dispersal across the oceans. Moreover, based on morphological observation and semi-section microscopy, the development pattern of IPC glander trichomes was described, and their physiological functions were also discussed.

## Introduction

Climate changes and land degradation are reducing crop production with the potential to threaten human substantial human development. During the last decades, plant genetics have offered concreted opportunities to increase yield and crop adaptability (Cortés and López-Hernández, 2021). According to the novel concepts reviewed by Cortés et al. (2021), crop wild relatives (CWR) are the potential reservoir of unique alleles at genes for abiotic tolerance. As the closest CWR of sweet potato, *Ipomoea pes-caprae* (L.) (IPC) has attracted the attention of the researchers. IPC belonging to the genus of Ipomoea under the family of Convolvulaceae is widely distributed in the littoral areas of tropical and subtropical regions of the world. Compared to the selection during domestication, natural adaptation has selected a broad spectrum of abiotic resistance alleles because of the wide-range variation of inhabited environments for the wild species. As we know, the coastal areas are windy and salty with high-temperature and high irradiation in summer. IPC species should have evolved strong adaption to those harsh conditions. It has been reported that IPC species could tolerate salinity as high as 225 mM NaCl (Suarez, 2011). However, it appears to be more susceptible to drought as compared with salinity (Zheng et al., 2018). Even though the routine test of IPC to temperature extremes has not been reported so far, Cheng et al. found that IPC could grow normally under natural temperature extremes (12°C and 42°C), and the underlying mechanisms have been deciphered in the insight of transcriptome analysis (Liu et al., 2020; Cheng et al., 2021).

In addition to the biological value, IPC’s ecological value also interests the researchers. IPC is a vine plant whose most extended branch can grow 20 meters in sandy soil (Martins et al., 2012; Miryeganeh et al., 2014). IPC can not only creep over the barren soil by high nutrition uptake with stoloniferous stems but also has developed root systems and various abiotic or biotic resistance. It is regarded as one of the potential pioneer plants for ecological improvement (Zhang et al., 2018; Bennett and Sherratt, 2019; Cheng et al., 2021). Hence, IPC has been used as the first “green barrier” for sand fixation, island-greening, and the ecological restoration of coastal regions and islands and, reef construction. Current studies have confirmed that IPC can absorb various types of heavy metals from salty soil and stored in the flowers, leaves, and stems (Baker, 1981; Kozak et al., 2015). IPC is selected as an indicator to assess the presence of selected elements (Baker, 1981). IPC has also been widely used as a medicinal plant. In China, Australia, Mexico, Thailand, and Brazil, IPC was usually used as a traditional medicine against several diseases, such as pain (Krogh et al., 1999; Rogers et al., 2000), ulcers, strains, wounds (Manigaunha et al., 2011), cancer (Pereda-Miranda et al., 2005; Manigauha et al., 2015), and inflammatory conditions (Rheumatoid arthritis, Ankylosing spondylitis, Osteoarthritis, Gout) (Venkataraman et al., 2013). The antinociceptive (Krogh et al., 1999), anti-inflammatory (Pongprayoon et al., 1991), antioxidant activities (Kodali and Sen, 2008), antispasmodic activity (Pongprayoon et al., 1992), antibacterial activity (Kumar et al., 2014), immunostimulatory activity (Philippi et al., 2010), and collagenase inhibitory activity (Teramachi et al., 2005) of IPC has already been scientifically proven. Although the biological, ecological and medicinal values of IPC have attracted the attention of researchers, the fundamental biological and genetic characteristics of IPC still remain unknown.

IPC has been considered one of the extraordinarily wide-distributed single species of land plants (Miryeganeh et al., 2014). It has been demonstrated that the genetic divergence of IPC subspecies located in different territories is very low, indicating the frequent gene exchanges of this species (Miryeganeh et al., 2014). While the pollen dispersal *via* insects or pollinators could enhance the gene flow between the subspecies, when two adjacent but distant populations are separated by hundreds of kilometres of the ocean (for example, between the main island and an oceanic island), it is less likely that the gene-flow could be mediated by the pollen dispersal (Nathan et al., 2008). Therefore, how IPC is widely distributed on tropical and subtropical coasts around the globe is very thought-provoking. Drifting *via* water is an important mechanical mechanism for dispersing seeds, the unique sexual reproductive organ of angiosperms. Long-distance dispersal (LDD) by water flow is the most effective way for the seeds. For a long time, people have been aware of the importance of LDD by seawater (Nakanishi, 1988). For example, most mangroves have seeds or propagules that can drift on seawater and be transported by ocean currents (Miryeganeh et al., 2014). Because of its effectiveness across long distances, species that exhibit sea dispersal have vast ranges (Tomlinson, 2016). IPC belongs to a group of pantropical plants, including a few other species from divergent families, such as *Canavalia rosea* Sweet. (Fabaceae), *Vigna marina* (Burm.) Merr. - *Vigna luteola* (Jacq.) Benth. (Fabaceae), and *Hibiscus tiliaceus* L. - *Hibiscus pernambucensis* Arruda (Malvaceae). Members of this plant group can use ocean currents as a vector to disperse their seeds over very long distances (Takayama et al., 2006; Takayama et al., 2008; Vatanparast et al., 2011). It has been reported that the seeds of IPC could be delivered more than 100 km away through LDD (Miryeganeh et al., 2014).

The seeds are inhouse the next-generation individuals, and the typical characteristic of angiosperms is that their seeds are enclosed in pericarps, which are critical for population expansion. During fruit development, the seeds inside develop and mature, which is a critical process connecting the previous generation of plants to the next generation. In the life cycle of plants, multicellular and unicellular processes alternatively occur in different tissues or reproductive units. Gametogenesis, the transformation from plant individuals to gametes, is the process of uni-cellularization in plants. However, seeds are sexual reproductive organs developed from ovules containing fertilized eggs, which are sexual reproductive units that undergo multi-cellularization to give rise to embryos. This process is called embryogenesis (Imbert, 2002). Seeds are also the basic unit of population dispersal, and seed coats can maintain the activity of the embryo during the period from seed maturation to germination under appropriate conditions (Penfield, 2017).

Sea dispersal of seeds promotes population spreading, the flow of individuals between populations, the colonization of unoccupied habitats, and the assembly of local communities from the metacommunity (Levine and Murrell, 2003; Levin et al., 2003). Dormancy and germination are the essential traits in the plant life cycle involved in species survival and offspring proliferation. Under suitable conditions, water is often the activator of seed germination, breaking seed dormancy, and then cotyledon and root breakthrough seed coat to complete the germination process (Seale and Nakayama, 2020). However, the effectiveness of LDD of seeds must be supported by long dormancy periods before they become widespread in the ocean. In this way, populations can exchange individuals and allow for the flow of genes between distant populations where pollen cannot migrate (Vatanparast et al., 2011). Dispersal to the ocean by drifting seeds would be the only possible way to maintain migration between these populations. Nakanishi (1988) has reported that the seeds of IPC species can float in seawater for up to 90 days and sustain high activity (Nakanishi, 1988). However, the biological characteristics adapting to the LLD of IPC were rarely reported.

Considering IPC’s biological, ecological and medicinal values, this study aims to investigate its genome size and chromosome numbers, providing the essential information for further genome deciphering work. Since the seeds are the dispersal unit for population expansion, we hypothesized that the seed structure and dormancy should fulfil the LLD. At the same time, the germination and early seedling development should be adapted to its habitat environment. In this sense, we extensively characterized fruit and seed developments, seed morphology, and seed germination in IPC. The results provided insights into the mechanisms of LDD of IPC seeds, which also explain the success in the population expansion of this species.

## Materials and methods

### Plant materials

The *Ipomoea pes-caprae* L. plants used for this study were naturally growing on the beach located in Changle, Fuzhou (Latitude 25°, 54’, 33˝N, Longitude 119°, 40’, 42˝ E), Fujian, China. During autumn, the newly matured brown seeds were collected directly from the wild-grown plants. Fruits were carefully selected prior to the release of the seeds. The seed germination analysis was conducted in the laboratory.

### Flow cytometry assay

To explore the appropriate gene size of IPC, flow cytometry was used to measure the DAPI-fluorescence intensity of the digested nuclei of IPC leaves using tomato and rice leaves as references. The 20 mg of leaf tissues were chopped with a very sharp razor in 1 ml ice-cold nuclei isolation (45 mM MgCl2·6H2O, 20 mM MOPS, 30 mM Sodium Citrate, 1% (W/V) PVP-40, 0.2%(v/v) Triton X-100, 10 mM Na2EDTA, and 20 μL/mL β-Mercaptoethanol, pH 7.0) (Loureiro et al., 2006). The homogenated samples were then filtered through a 42-mm nylon mesh, and 10 ul of DNA fluorochrome solution (50 mg/ml PI, 50 mg/ml RNase) was added to the flow-through for staining the nuclei. After 5 minutes of incubation at room temperature, the nuclei solutions were subject to FLC analysis on a BD FACScalibur Flow cytometry platform (Cheng et al., 2019).

### Chromosome spreading and chromosome number analysis

The pollen grains with 0.3–0.5 mm diameter were collected and used for chromosome number observation. The chromosome spreads of microspores were prepared as described previously (Ross et al., 1996) and stained with 1.5 μg/ml 4,6-diamidino-2-phenylindole (DAPI) before microscopy. Images of chromosome spreads were taken with a Zeiss (Model) microscope following the method reported by Cheng et al. (2019).

### Seed floating, germination, and growth conditions

The newly matured seeds harvested as mentioned above were used for the floating test. 100 seeds were soaked in water for 24 hours at room temperature for 24 hours and then subjected to observe their floating potential. The seeds harvested from the wild-grown IPC were used for the germination test. A pair of scissors was used to carefully cut down the seed corners without hurting the embryo inside to break down the seed coats (scarified seeds). The intact or scarifeid freshly harvested (FH) and six months after ripening (AR) seeds were soaked in the water and placed in a 37°C incubator to test the imbibition rate and germination rate. Three replicates with 150 seeds for each treatment, the imbibition and germination rates were calculated, and the difference significances were estimated using the t-test method. The germinated seedlings were transformed into the soil and planted in a 25°C greenhouse with 16 light/8 dark photoperiods, 200 μmol·m ^–2^·s ^–1^ light intensity, and 30%–50% relative humidity. The plant morphology photographs were taken at 24, 48, and 72 hours after germination (HAG) and 4, 5, 6, 7, 10, 12, 14, 16, 18, 20, 22, and 24 days after germination (DAG) *via* a Nikon D7200 digital camera.

### Photographing and SEM microscopy

The images of seed germination and embryo development were photographed by a Nikon D7200 digital camera. The images of the seed surface and glandular trichomes were taken with a scanning electron microscope (SEM, TM3030 PLUS).

### Semi-thin section examination

The stem apical meristems (SAMs) and the true leaves at 1 to 6 DAGs were collected and fixed in FAA (50% ethanol, 5% (v/v) acetic acid, 3.7% (v/v) formaldehyde) for at least 24 hours in the dark (Zhao et al., 2021). The fixed samples were then dehydrated gradually by treatment in sequential ethanol solutions with a gradient concentration of 30%, 50%, 70%, 80%, 90%, 95%, and 100%, and each treatment lasted 20 minutes. The dehydrated samples were immersed in acetone solution for 20 minutes twice and finally embedded in Eponate-12 resin (Ted Pella, Redding, CA, USA) (Cai et al., 2017). Sections of 3-micrometer thickness were cut using a Leica Ultracut Sultramicrotome and stained with 0.1% toluidine blue before imaging with a BX63 microscope (Olympus) under bright-field optics.

## Results

### The genome size of IPC is approximately 1.08 GB

**Figure 1:**
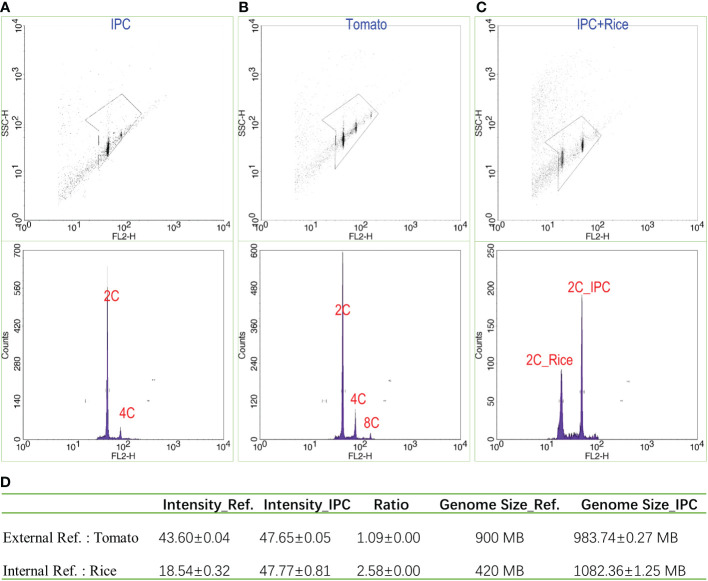
The estimation of IPC genome size by flow cytometry. **(A)** The flow cytometry result of IPC; **(B)** The flow cytometry result of tomato; **(C)** The flow cytometry results of IPC using Rice as the internal reference. **(D)** The genome sizes of IPC were estimated by flow cytometry using Tomato or Rice as references. SSC-H: The height volume of the Side Scatter indicating the complexity of the particle; FL2-H: The fluorescence intensity (height) detected by the FL2 channel. Counts; the counts of nuclei detected.

The IPC nuclei showed significant 2C and 4C peaks with fluorescence intensities of 47.65 and 65.30, respectively (**Figure 1A**). Meanwhile, the digested nuclei of tomato leaves were also subjected to flow cytometry analysis as an external reference, resulting in fluorescence intensities of 43.60 for 2C and 87.20 for 4C (**Figure 1B**). The 2C fluorescence intensity ratio of IPC to Tomato is 1.09. Considering the genome size of the tomato is 900 MB (Sato et al., 2012), it is estimated that the genome size of IPC is approximately 983.74 ± 0.27 MB. To accurately estimate the genome size of IPC, it is better to conduct the Flow cytometry assay with an internal reference. Since the genome sizes of IPC and tomata are very close, the digested nuclei of rice leaves were also used for this analysis. As shown in **Figure 1A**, the 2C fluorescence intensities of Rice and IPC were 18.54 ± 0.32 and 47.77 ± 0.81, respectively (**Figure 1C**). The fluorescence intensities ratio of IPC to Rice is around 2.58. The internal reference we used here was *Oryza sativa* Indica, and the average genome size of the assemblies of the *Oryza sativa* Indica group was approximately 420 MB (Yu et al., 2002). In this analysis, it is estimated that the genome size of IPC is approximately 1.08 GB (**Figure 1D**).

### The chromosome number analysis of IPC

**Figure 2:**
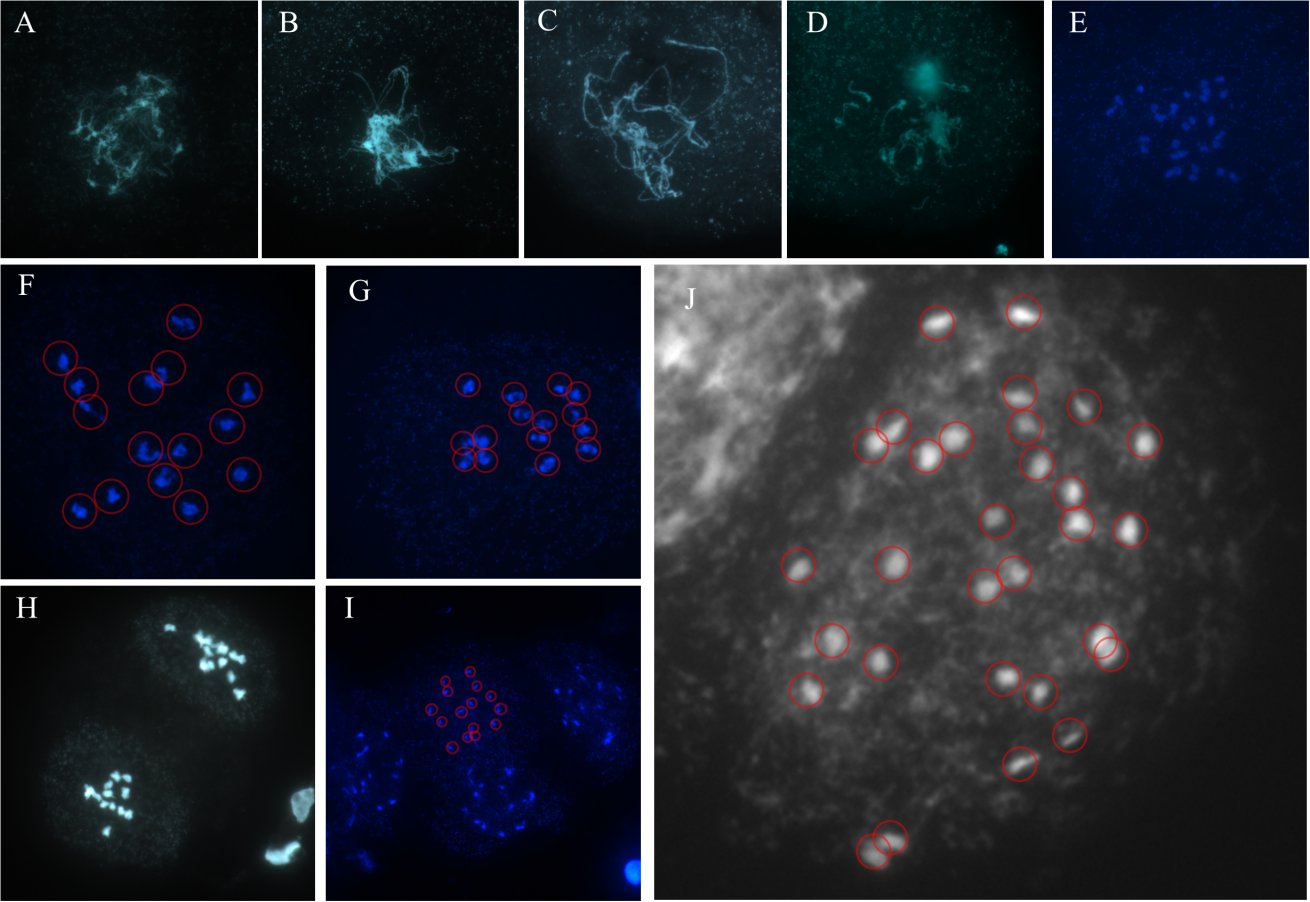
The meiosis chromosome behavior during IPC pollen development. **(A–I)** The pollens were dyed with DAPI, and the samples were mounted on the slides for fluorescence microscopy. The figures showed the chromosomes at Leptotene **(A)**, Zygotene **(B)**, Pachytene **(C)**, Diplotene **(D)**, Diakinesis **(E)**, Prophase I **(F)**, Metaphase I **(G)**. Metaphase II **(H)**, and Telophase II **(I)**. **(J)** The mitosis chromosomes indicate the chromosome number of IPC.

A set of images showed a complete meiosis process of IPC, exhibiting the chromosome behaviours at Leptotene, Zygotene, Pachytene, Diplotene, Diakinesis, Prophase I, Metaphase I. Metaphase II, and Telophase II phases (**Figures 2A–I**). The haploid IPC chromosome number is 15. Meanwhile, the chromosome count of a somatic cell at mitosis metaphase was 30 (**Figure 2J**). It is concluded that the chromosome number of IPC species investigated in this study is 2n = 30.

### The development of IPC’s fruit, seed, and embryo

**Figure 3:**
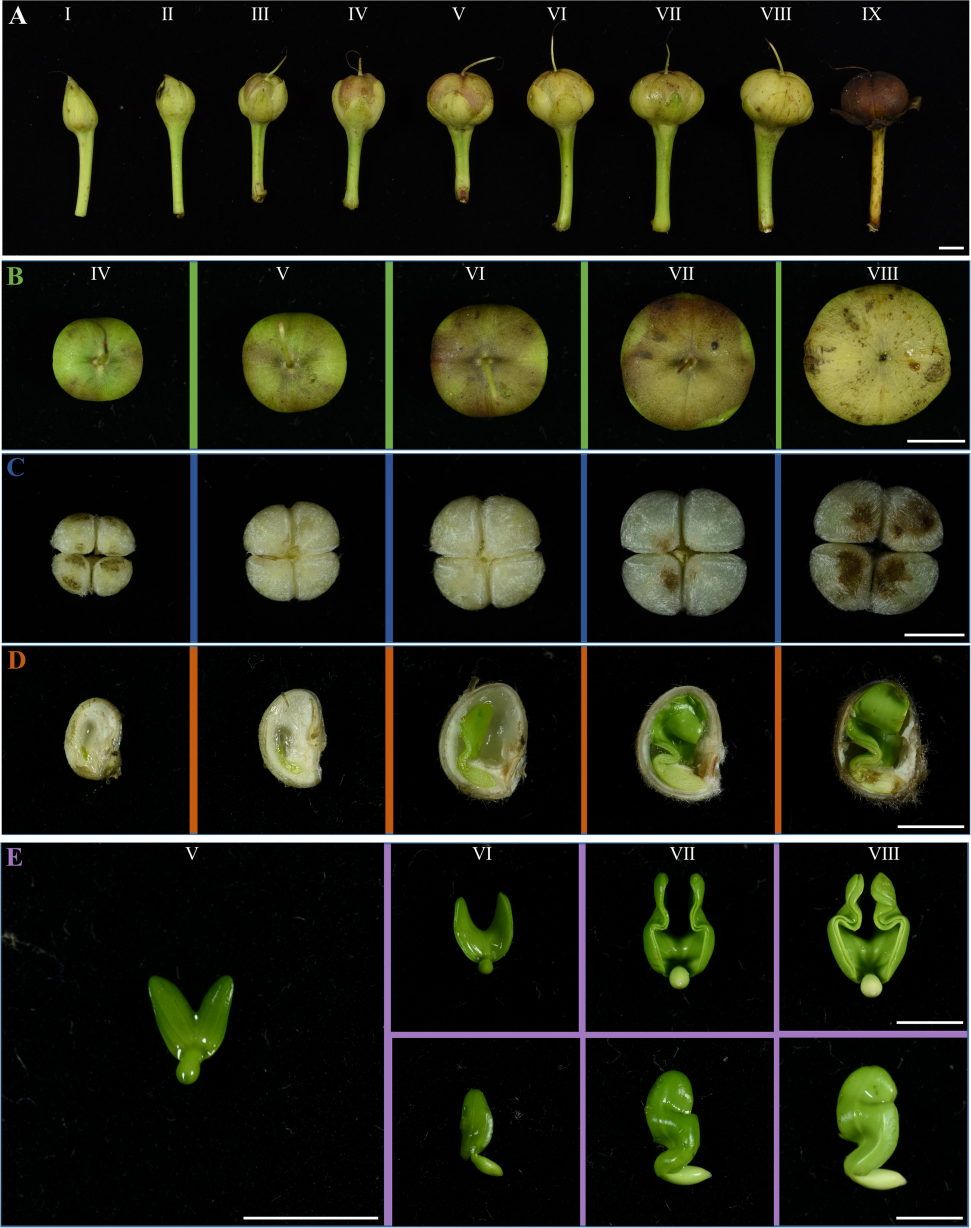
The fruit, seed, and embryo development of IPC. **(A)** The IPC fruits at I-IX stages showed the fruit development of IPC. The fruits are developed from the compound pistils of connate carpels, with two locules containing two seeds. Bar = 0.5 cm. **(B)** Figures show the top view of fruits at IV-VIII stages. Bar = 0.5 cm. **(C)** Figures show the IPC seeds at IV- VIII stages. Bar = 0.5 cm. **(D)** The vertical section images of IPC seeds at IV-VIII stages. Bar = 0.5 cm. **(E)** Images show the IPC embryos at V-VIII stages. Bar = 0.5 cm.

The development of IPC fruit was categorized into nine development stages (**Figure 3A**). The fruits at stages I and II are entirely enclosed by calyxes. With the growth of the seed, the calyx also gradually grew. The calyxes are dry and withered until stage IX, and the IPC seeds mature. At stage V, the fruit shapes are grown to the final shape, and the fruit size continues to increase. From stage V to IX, the *IPC* fruits and seeds are changed in size and are unchanged in shape along their development process. The IPC fruits are developed from a compound pistil of connate carpels, with two locules containing two seeds each. Moreover, the fruits have long axes, short axes, and two seeds in a locule separated by a septum. At the IX developmental stage of the fruit, the fruit splits from the central axis along the septum at maturity. The type of IPC placenta is axial placentation (**Figures 3B, C**).

The seeds from stages IV to VIII were cut into two halves to observe the development of embryos inside (**Figure 3D**). In the early stages of embryo development (Stages V and VI), the milky white and transparent endosperms occupied most of the space in the seed coats. At this time, the embryo is tiny, and the two cotyledons are heart-shaped and overlapped, existing at the micropyle end of the ovule. With the degeneration of endosperm and embryo growth from stage V, the cavity in the seed expands rapidly. In the VI stage, the seed reaches its maximum size, and a thick seed coat is formed, enclosing the embryo with apparent cavities. In the VII stage, the cotyledons reached their maximum size and were folded because of space limitations (**Figure 3E**). At this stage, the cavity of the seed reaches maximum. From the VII stage, the villus (tomenta) on the top of the seed began to turn brown from white, and the embryo’s growth gradually stopped (**Figure 3D**). To the IX stage of fruit development, with the dehydration of the fruit and seed, the pericarp turned brown, the seed coat turned black, and the cotyledons of the embryo finally turned milky yellow (**Figures S1A–D**).

### Seed floatation and seed dormancy of IPC

**Figure 4:**
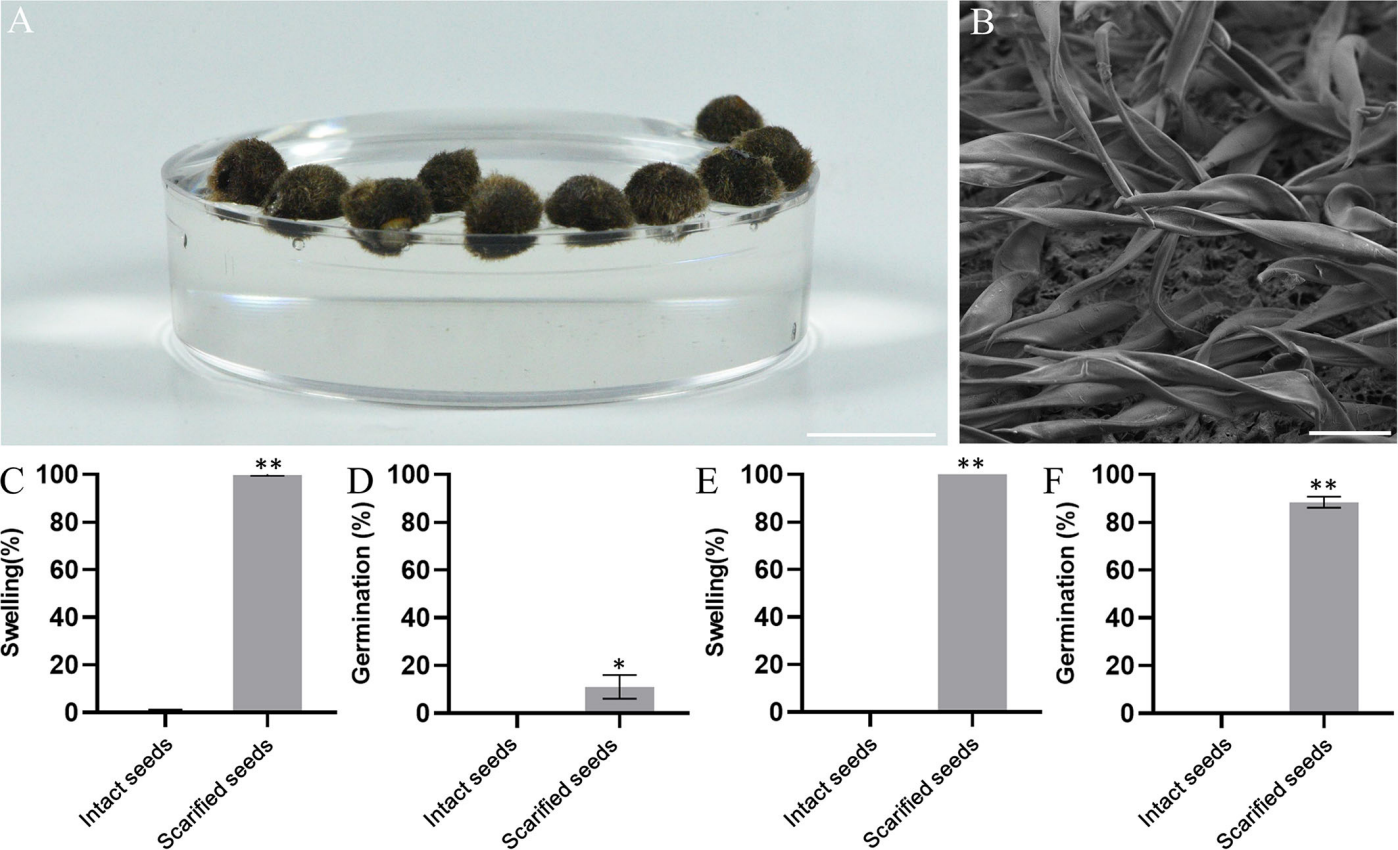
Morphology and dormancy of IPC seeds. **(A)** The IPC seeds float in the water easily. Bar=1 cm. **(B)** Image shows the surface of a mature IPC seed. Bar= 100 μm. **(C)** Bars show the imbibition rate of intact and scarified freshly harvested (FH) IPC seeds. **(D)** Bars show the germination rate of intact and scarified FH IPC seeds. **(E)** Bars show the imbibition rate of intact and scarified six months after ripening (AR) IPC seeds. **(F)** Bars show the germination rate of intact and scarified AR IPC seeds. Error bars indicate the standard deviation, ** indicates the P < 0.01 significant difference evaluated by t-test.

To uncover the secret of long-distance disposal of IPC, the dry IPC seeds were put into water to test their floatage. As shown in **Figure 4A**, all IPC seeds were floating on the water, with approximately two-thirds above the water. One reason might be the formation of cavities during seed development. Another secret is the hydrophobicity of the IPC seed surface. Under SEM, we observed that the seed coats of mature IPC seeds are covered by tomenta, which are in lamellar strips shape and right-handed spiralled (**Figure 4B**). The dense tomenta provide strong hydrophobicity to the IPC seeds, giving rise to the potential of the IPC seeds drifting over long distances.

For the freshly harvested (FH) seeds, 99.78% of the scarified seeds were swelled after several hours of soaking in water, while only a few intact seeds (0.67%) were swelled, and for the six months after-ripening (AR) seeds, the rates were 100% and 0.22% (**Figure 4E**; **Table S2**). The non-swelling state of the intact seeds from both experimental groups (FH and AR) was maintained for over six months soaking in the water, indicating that the hard seed coat of mature IPC seed could prevent the imbibition of seeds with high efficiency. The germination rates of the FH and AR IPC seeds were also tested, and only 11.11% of the FH scarified seeds germinated. However, a much more significant proportion of AF scarified seeds (88.59%) germinated, indicating that the mature IPC seeds have significant dormancy even under the appropriate germination conditions (**Figures 4E, F**; **Table S2**).

### Seed germination and SAM development of IPC

**Figure 5:**
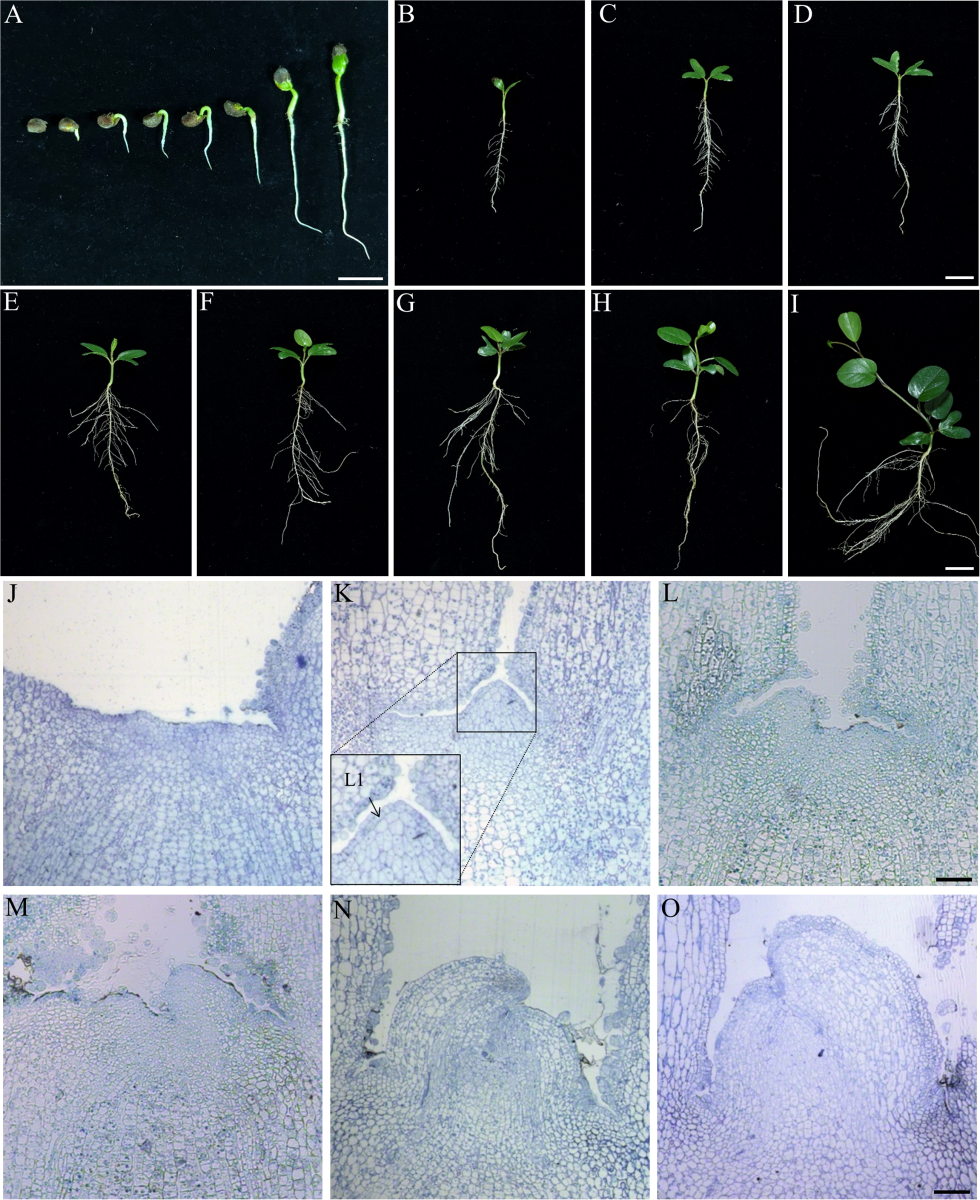
Seed germination and stem apical meristem development of IPC. **(A)** The epigeal germination pattern of IPC seeds. Figures show the seedlings at 24, 48, and 72 hours and 4, 5, 6, and 7 days after germination. Bar = 2 cm. **(B–I)** Figures show the IPC plantlets at 10, 12, 14, 16, 18, 20, 22, and 24 days after germination. Bar = 2 cm. **(J–O)** Semithin section images of stem apical meristems from 1 to 6 days after germination show the development of IPC SAM. Bar = 500 μm.

Twenty-four hours after imbibition, The roots break through the seed shell first, and their growth is more predominant when the hypocotyledons elongate slowly from day one to day four. Cotyledons and stems synchronously grow rapidly from the fifth day to the seventh, while taproots begin to form lateral roots (**Figure 5A**). The IPC plantlets development at 10, 12, 14, 16, 18, 20, 22, and 24 days after germination were shown in **Figures 5B–I**. The phyllotaxis of IPC are alternative, and there is a thick leathery surface on the leaves to prevent water loss. The apex of the leaf blade is dented or lobed, shaped like a saddle, which might be the origin of its Chinese alias “Maan Teng.” The observation of IPC germination also indicated that the seed germination pattern of IPC belongs to epigeal germination (**Figure 5A**).

To determine the formation pattern of IPC true leaves, the development of SAM was observed on the germinating seeds at stages of 24, 48, 72 h, and 4, 5, and 6 days after germination (**Figures 5J–O**). It was found that the morphology of the stem apical meristem of IPC was still flat at 24 h after germination (**Figures 5J, K**), and the SAM showed an apparent tunica-corpus structure at 48 h after germination (**Figure 5K**), in which we could see the tunica composed of one layer of cells, and the initial layer of the corpus at the second layer composed of cells with ununiformed sizes. The leaf primordium was formed at 72 h of germination (**Figure 5L**).

### The morphology and development of glandular trichomes

Our observation showed that the GTs are mainly presented on the surfaces of the younger leaves (**Figure S2A**). The adaxial surfaces have a much higher density than the abaxial surfaces (**Figures S2B, C, E, F**). Most of the GTs on the abaxial surfaces were broken, probably because of the degradation during secreting sticky substances to protect the young leaves during the early development stage. However, few GTs were observed on the surface of the elder leaves rather than the GT vestiges (**Figures S2D, G**).

**Figure 6:**
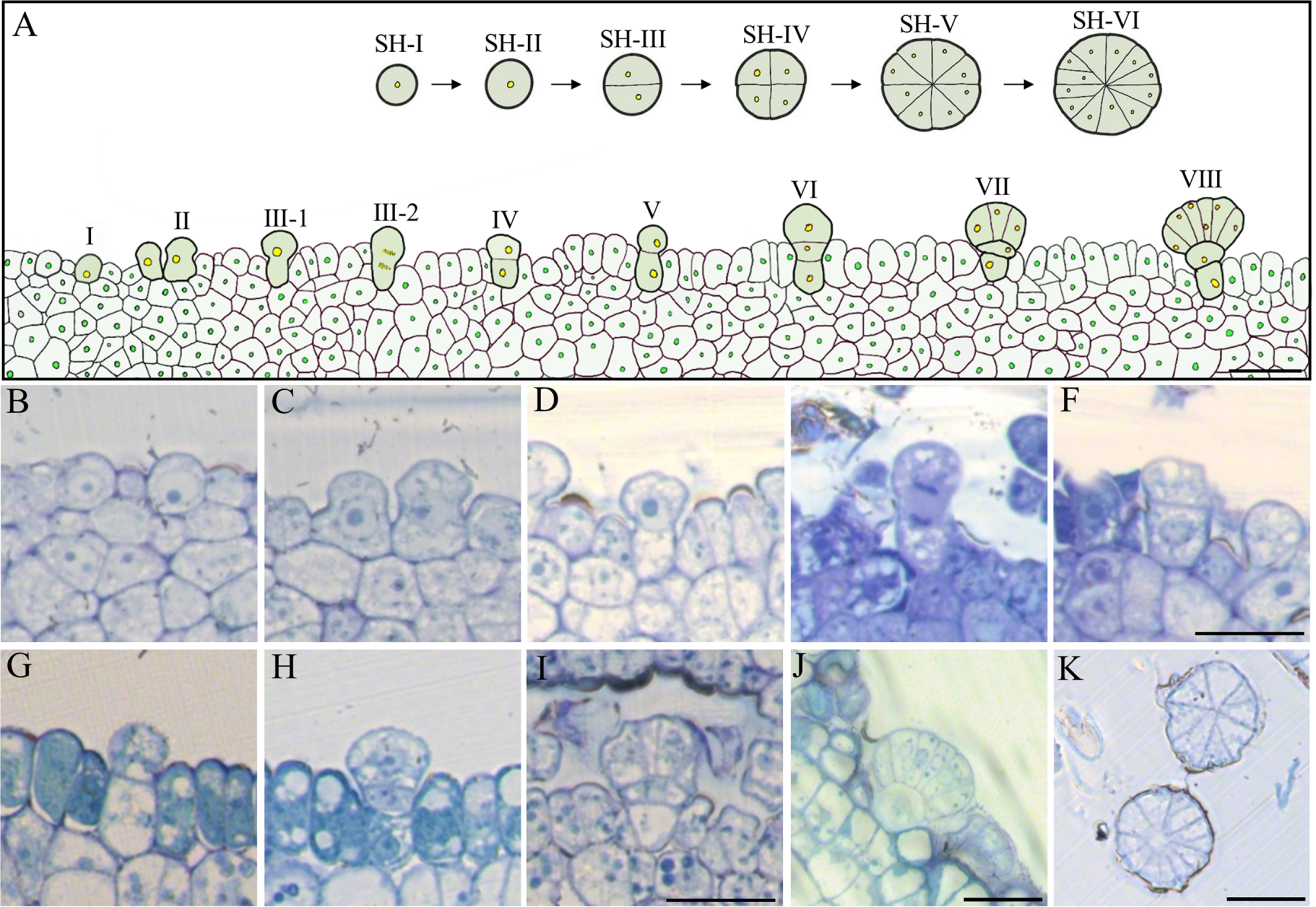
Histogenesis of the epidermal GT in the early stage of IPC. **(A)** Pattern diagram of GT development. Bar= 250 μm. **(B–J)** Semithin section (vertical) images showed the development process of IPC GT, b (I), c (II), d (III-1), e (III-2), f (IV), g (V), h (VI), I (VII), j (VIII). Bar = 250 μm. **(K)** The transverse section images of IPC GT six days after germination. Bar= 250 μm. SH, Secretory Head.

Kuster et al. (2016) histologically observed the morphology of GT in IPC living in Restinga, Brazil, which is multicellular and composed of a secretory head, a stalk, and a basal cell (Kuster et al., 2016). In this study, we have also observed the typical multicellular structure of GT in IPC living in Changle, China. To understand the developmental process of the multicellular GT of IPC, we constructed the histogenesis patterns of IPC glandular trichomes based on the section images of the GT at different developmental stages (**Figure 6A**). At the beginning of germination, the GTs could be observed on the surface of the IPC leaf blade. At 72 h after germination, the number of GT increased significantly. The GTs originated from the epidermal cells (**Figure 6B**, stage I). The epidermal cell obtained the GT destination first increases theirs lengthens (**Figure 6C**, stage II), and the top end of the elongated epidermal cell is expanded slightly (**Figure 6D**, stage III-1) and entering the first mitosis resulting in a bicellular-trichome structure containing two daughter cells (**Figures 6E, F**, stageIII-2, IV). The one internal of the epidermal develops into the basal cell later (**Figure 6G**, stage V), and another on top of the basal cell undergoes the second division and forms apical and basal cells (**Figure 6H**, stage VI). The basal cell does not expand much and develops to the stalk of the GT (**Figure 6I**, stage VII), while the apical cell increases in size and forms the globular-shaped secretory head of GT, containing eight to twelve cells (**Figures 6J, K**, stage VIII). The division of the apical cells is almost symmetrical, and the division pattern is shown in the upper right part of **Figure 6A** (stage SH-I to stage SH-VI) (Smaoui et al., 2011; Shabala et al., 2014).

## Discussion

Increasing climate change threatens food production and, thus, human survival and development, among which the impact of global warming is significant. In terms of the influence of high temperature on crop yield, high night temperature is more significant, which has been proven as a key proxy for heat damage, together with dry stigmas and unviable pollen (López-Hernández and Cortés, 2019; Burbano et al., 2021; Abrol and Ingram, 2022). The abiotic stresses, especially salt and drought, shared a common regulatory system and cross-talk in stress perception, signal transduction and downstream response (Cortes et al., 2013). For instance, MAPK cascades and the cross-talk between ABA signalling and biotic signalling were extensively reviewed (Huang et al., 2012). Osmotic regulation due to salt and drought stress may be pleiotropic between both complex traits (Cortés et al., 2012; Cortés et al., 2012), compared to stomata regulation (Blair et al., 2016). However, The CWRs have evolved a vast range of biotic alleles which may surpass the conventional cultivars during natural selection, and those alleles are precious gene resources for crop breeding (Khoury et al., 2016; Cortés et al., 2021), especially in coping with climate changes (Cortés and López-Hernández, 2021). A successful accomplishment has been made in the cropping practice of common beans, in which the last-generation genome-environment association has revealed the genetic basis of heat and drought tolerance (Cortés et al., 2012; López-Hernández and Cortés, 2019), which has been successfully applied in the genetic improvement of common bean targeting to enhancing drought and salt tolerance (Burbano et al., 2021). IPC species are the CWRs of sweet potatoes, and their biological and genetic investigation is of great significance to the abiotic tolerance improvement of the latter.

### The genome size and chromosome number analyses of IPC provide fundamental information for further genome deciphering

The genome contains all the genetic information of the organism, and the genome sequence is the complete list of the nucleotides (A, C, G, and T for DNA genomes) that make up all the chromosomes of an individual or a species. Genome size is the fundamental characteristic of the specific organism, which is also an essential parameter for evolutional and taxonomic analyses (Bainard et al., 2020). It has been reported that the genetic diversity among the IPC subspecies inhabited on different continents is very low because of their frequent gene exchanges (Miryeganeh et al., 2014). However, the genome size of IPC species is unknown till now. The flow cytometry analysis in this study showed that the genome size of IPC is approximately 1.08 GB. The chromosomes are the organizer of the genetic information for a specific species, and the chromosome number is another fundamental characteristic of a plant species, which is usually used as the identification parameter for species and varieties. Even though the chromosome number information has been reported in the previous publications, the exact chromosome number of IPC, especially for the IPC habited in Changle, is unknown. To this end, the chromosome behaviour during the Meiosis of IPC male gametophyte and the chromosome counting analysis during mitosis were investigated in this study. The results showed that the chromosome number of IPC is 2n=30. The genome size and chromosome number analyses of IPC will provide fundamental information for further genome deciphering.

### The seed morphology and germination of IPC facilitate the long-distance dispersal by seawater

Since most land plants have sessile life forms, seed dispersal is important for population expansion and subspecies migration. The migration of subspecies exchanges individuals and keep gene flow between distant populations (Cain et al., 2000; Levin et al., 2003; Nathan et al., 2008). In most cases, the plants appear to act as passive units whose seeds or other diaspores are transported far away from the parent plants by different vectors, such as animals, wind, and water, at a given time (Nathan, 2006; Nathan et al., 2008). During evolution, plants have evolved various strategies to increase their seed dispersal in the following ways: flight mechanisms, animal transportation, and explosive spreading. However, those dispersal mechanisms rely on the formation of specialized anatomical and morphological adaptations for using physical or biological dispersal vectors (Seale and Nakayama, 2020). For example, the plants of Asteraceae and the other 17 plant families produce diaspore heteromorphism to facilitate seed dispersal through wind, animal, and water (Imbert, 2002; Seale and Nakayama, 2020). Because of the long-distance dispersal (LDD), IPC plants are distributed worldwide in subtropical and tropical coastal areas, with frequent transoceanic gene exchange (Li et al., 2019). Previous studies have investigated their adaption to salt stress and temperature extremes in transcriptomic levels (Wood et al., 2020; Cheng et al., 2021). In this study, the IPC seeds were extensively investigated in aspects of morphology, anatomy, germination, and dormancy, trying to decipher the mechanical mechanism of IPC to facilitate the LDD and explain why IPC is one of the most widely distributed plants in the world. Our results showed that IPC seeds develop hard and thick seed coats covering massive hydrophobic tomenta that give seeds floating potential in water (**Figure 4A**). Seed drifting is the most important factor for IPC plants to pass through ocean circulation (Takayama et al., 2006; Takayama et al., 2008; Vatanparast et al., 2011; Miryeganeh et al., 2014). On the other hand, the newly-harvest IPC seed would not germinate even with scarification (**Figure 4**). As we know, water and air are the main conditions for seed germination. The IPC seed coats are very hard, preventing the water from getting into the seed to stimulate germination. Our results show that the hard, dense seed shell of IPC plays a vital role in preventing air and water from entering the seed interior. The seeds that are broken in the seed coat can quickly emerge from dormancy and begin the germination stage in suitable conditions. The comparison of germination rates between FS and AS indicated that the seeds of IPC need to undergo late ripening or a certain dormant period before they can be germinated. In summary, the specialized seed coats of IPC help the seed float on the water. Meanwhile, the hard seed coats act as a physical barrier to prevent seed imbibition, and the dormancy of mature embryos acts as a physiological factor preventing the seeds from germination, thus giving the IPC seeds enough time for LLD over the sea.

### The adaption of IPC in the perspective of SAM and GT developments

Under a broad range of stressful conditions, such as high irradiation, high temperatures, increased salinity, and water shortage, plants may have been driven by the development of a set of morphological, anatomical, and physiological adaptations. Shoot apical meristem (SAM) and root apical meristem (RAM) are formed under the activity of the stem apical meristem to complete the complex life process. It is believed that leaves are formed after seeds germinate when it comes to leaf formation. In many plants, however, vegetative leaves were formed during seed development. For example, the well-known monocotyledons maize and wheat, dicotyledons soybean, or the three euphylla of Rice start during seed development, while cucumber seeds do not have prominent vegetative leaves at maturity but only produce leaf primordia (Vernoud et al., 2005; Deng et al., 2021). In this analysis, the ture leaves were not observed in the IPC seeds and the SAM, and the ancestor of the true leaves became observable during seed germination. The inactivation or the later maturation of SAM of the embryo might be one of the physiological mechanisms of IPC seeds to protect the meristem cell lineage.

In some plant species, secretory structures like laticifers and nectaries may play an essential role in plant survival (Kuster et al., 2016; Le Roux, 2009). Laticifers, for example, secrete terpenoids and caoutchoid that protect against herbivorous insects and microbes and seal wounds (Fahn, 1979). Previous studies have also observed the secretory structures, glander trichomes (GTs), on the surface of the leaves of IPC. However, the function of the IPC GTs has rarely been demonstrated. It has been observed that the glandular trichomes (GTs) were presented on both sides of the IPC leaf blade, secreting polysaccharides mucilage which plays essential roles in wound response, plant defense, and water maintenance (Pérez-De-Luque et al., 2006; Zimmermann et al., 2007; Fisher et al., 2009). Our observation showed that the GTs are mainly presented on the surfaces of the younger leaves (**Figure S2A**). Kuster et al. (2016) have observed that this kind GTs widely exists in Ipomoea species, such as *Ipomoae imperati* and *Ipomoae pes-caprae*, mainly distributed in the tissues such as leaves, petioles, and stems, which are rich in pectin and other polysaccharides (Kuster et al., 2016). The Ipomoea GTs can secrete fluid, but in which the sodium ions were not detected, indicating that these GTs are not salt glands. It is currently believed that the primary function of the GTs is to seal wounds, resist herbivores and prevent water loss (Zimmermann et al., 2007; Fisher et al., 2009). Based on our results, we found that IPC GTs might play an essential role in protecting seedlings and leaves. The secretory substance from the GT may form the leathery membranes on the mature leave. Under harsh coastal conditions with high temperatures, high radiation, high wind, and high salt content of the soil, the mature leaves of IPC were covered by a layer of the leathery membrane, which protects the leaves from irradiation and water loss under the coastal conditions. However, in young leaves, the sticky fluid secreted by GTs makes two halves of the leaf blades fold and stick together to reduce water transpiration before the leathery membrane formation (**Figure S2**). The GTs were observed as early as the seed germination. On the first day after germination, the completely developed glandular trichomes could be observed on the semi-section of the cotyledons and primordium. While on the third day after germination, more dense GTs were observed on the cotyledon than on the surfaces of mature leaves. It is also observed that almost all of the GTs degenerated on the mature leaves (**Figure S2**). Those observations indicated that the GTs might play an essential role in protecting IPC leaves, especially in preventing water loss. It has also been reported that the viscous fluid secreted by GTs is mainly composed of hydrophilic polysaccharides, which the researchers speculated might protect and lubricate the meristem during early development (Fahn, 1979; Martins et al., 2012; Kuster et al., 2016). This is consistent with our observation of a large number of glands in the apical meristem.

## Data availability statement

The original contributions presented in the study are included in the article/**Supplementary Material**. Further inquiries can be directed to the corresponding authors.

## Author contributions

YC and YQ conceived and designed the research. KY, BH, CD, JS, ZL, and FD performed the experiments. LW, CP, and MA helped with a critical discussion on the work. YC and KY wrote the manuscript. MA and YQ revised the manuscript. All authors discussed the results and approved the final version of the manuscript.
