# Supplementary material for: The genome size, chromosome number and the seed adaption to long-distance dispersal of Ipomoea pes-caprae (L.)
Source: Front Plant Sci. 2023 Mar 2;14:1074935. doi: 10.3389/fpls.2023.1074935 (PMC10017971; doi:10.3389/fpls.2023.1074935)
Supplement: Supplementary file 1 [file DataSheet_1.pdf]

**Supplementary Figures and legends**

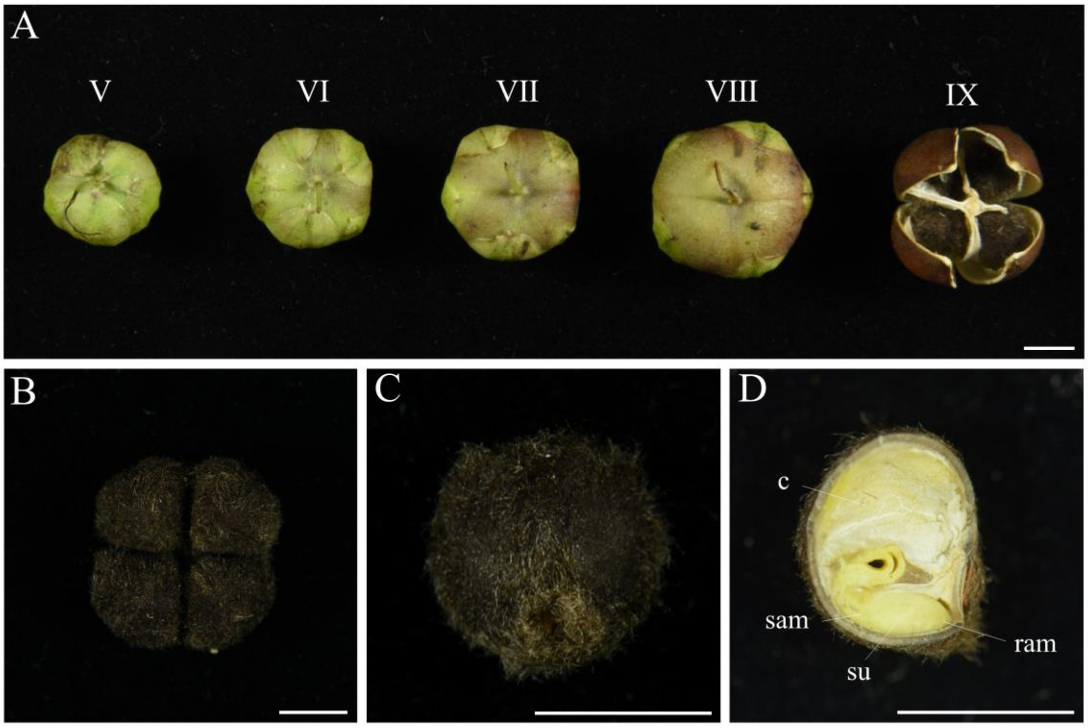


**Figure S1. Fruit and seed morphologies of IPC. (A)** Figures show the top view of fruits at IV-VIII stages. Bar = 0.5 cm. **(B,C)** Figures show the IPC mature seeds. Bar = 0.5 cm. **(D)** Images show the a dissected seed. Bar = 0.5 cm.

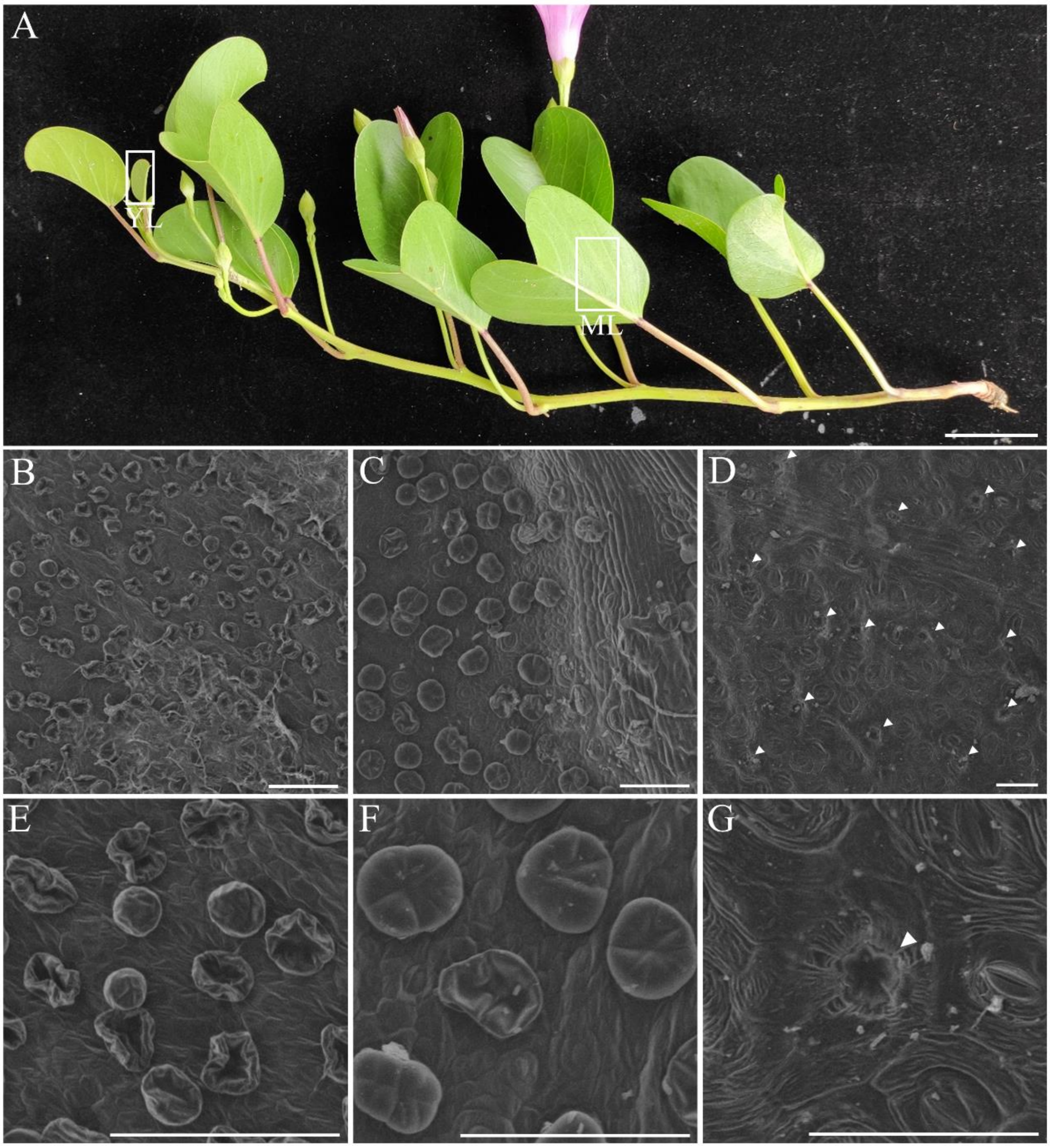


**Figure S2. The glandular trichomes on the surfaces of young and mature leaves. (A)** A flowering branch of IPC showed the leaves and floral inflorescence. **(B, E)** The GTs on the adaxial epidermis of IPC young leaf. **(C, F)** The GTs on the abaxial epidermis of IPC young leaf. **(D, G)** The degraded GTs on the abaxial epidermis of the IPC mature leaf. (YL, young leaf; ML, mature leaf; Bar for A = 2 cm; Bars for B-G = 100 um.)

**Table S1. Estimation of IPC genome size by flow cytometry analysis.**

| | Replicates | Intensity Reference | Intensity IPC | Ratio | Genome Size_Ref | Genome Size_IPC |
|---|---|---|---|---|---|---|
| **External Reference: Tomato** | 1 | 43.63 | 47.69 | 1.09 | 900.00 | 983.75 |
| | 2 | 43.60 | 47.67 | 1.09 | 900.00 | 984.01 |
| | 3 | 43.56 | 47.60 | 1.09 | 900.00 | 983.47 |
| **Internal Reference: Rice** | 1 | 18.90 | 48.71 | 2.58 | 420.00 | 1082.44 |
| | 2 | 18.38 | 47.31 | 2.57 | 420.00 | 1081.08 |
| | 3 | 18.33 | 47.29 | 2.58 | 420.00 | 1083.57 |

**Table S2. The germination and dormancy of IPC seeds.**

| Seed type | Treatment | Swollen rate_Mean | Swollen rate_SD | Germinaiton rate_Mean | Germination rate_SD |
|---|---|---|---|---|---|
| FH seeds | Seed coats intact | 0.67% | 0.54% | 0.00% | 0.00% |
| | Seed coats broke down | 99.78% | 0.31% | 11.11% | 4.94% |
| AR seeds | Seed coats intact | 0.22% | 0.31% | 0.00% | 0.00% |
| | Seed coats broke down | 100.00% | 0.00% | 88.44% | 2.27% |
